# Ecological Consequences of Shifting the Timing of Burning Tallgrass Prairie

**DOI:** 10.1371/journal.pone.0103423

**Published:** 2014-07-31

**Authors:** E. Gene Towne, Joseph M. Craine

**Affiliations:** Division of Biology, Kansas State University, Manhattan, Kansas, United States of America; Helmholtz Centre for Environmental Research - UFZ, Germany

## Abstract

In the Kansas Flint Hills, grassland burning is conducted during a relatively narrow window because management recommendations for the past 40 years have been to burn only in late spring. Widespread prescribed burning within this restricted time frame frequently creates smoke management issues downwind. A potential remedy for the concentrated smoke production in late spring is to expand burning to times earlier in the year. Yet, previous research suggested that burning in winter or early spring reduces plant productivity and cattle weight gain while increasing the proportion of undesirable plant species. In order to better understand the ecological consequences of burning at different times of the year, plant production and species abundance were measured for 20 years on ungrazed watersheds burned annually in autumn, winter, or spring. We found that there were no significant differences in total grass production among the burns on either upland or lowland topographic positions, although spring burned watersheds had higher grass culm production and lower forb biomass than autumn and winter burned watersheds. Burning in autumn or winter broadened the window of grass productivity response to precipitation, which reduces susceptibility to mid-season drought. Burning in autumn or winter also increased the phenological range of species by promoting cool-season graminoids without a concomitant decrease in warm-season grasses, potentially widening the seasonal window of high-quality forage. Incorporating autumn and winter burns into the overall portfolio of tallgrass prairie management should increase the flexibility in managing grasslands, promote biodiversity, and minimize air quality issues caused by en masse late-spring burning with little negative consequences for cattle production.

## Introduction

Periodic burning is required for the maintenance of tallgrass prairie [1]. The responses of prairie vegetation to fire, however, can vary widely depending upon when the fires occur [2]. Management and conservation objectives such as biomass production, livestock performance, wildlife habitat, and control of specific plant species, often influence when grasslands are burned. In some prairie regions, timing of seasonal burns have been used to manipulate the balance of C_3_ and C_4_ species [3], control woody species [4], stimulate grass flowering [5], and alter the proportion of plant functional groups [6]. Most grassland fire research, however, has focused on either burn frequency or comparing growing season burns with dormant season burns, and there are few studies that differentiate effects from seasonal burning within the dormant season [2]. In the Kansas Flint Hills, when prairies are burned is an important management issue, but the ecological consequences of burning at different times are poorly understood.

The Flint Hills are one of the last remaining regions supporting extensive native tallgrass prairie in North America and frequent burning is integral to its preservation and economic utilization [7]–[9]. Since the early 1970’s, recommendations have been to burn Kansas Flint Hills grasslands annually in late spring, typically once the dominant grasses have emerged 1.25–5 cm above the soil surface [10]. Although frequent late-spring burning has maintained the Flint Hills grassland, the resultant smoke plumes from en masse burning often leads to air quality issues in nearby cities [11], [12]. Concentrated smoke from grass fires produce airborne particulates, volatile organic compounds, and nitrogen oxides that facilitate tropospheric ozone production [13]–[15]. Burning in late spring also generates more ozone than burning in winter or early spring due to the higher air temperatures and insolation [16]–[18].

If the Flint Hills tallgrass prairie, its economic utilization, and high air quality are all to be maintained, a good understanding of the consequences of burning at different times of the year is necessary. Burning earlier in spring has been regarded as undesirable because it putatively reduces total biomass production [19]–[21], increases cool-season graminoids and undesirable forbs [22], [23], is ineffective in controlling woody species [19], [22], [23], and lowers monthly weight gains of steers [23] compared to burning in late spring. Consequently, burning exclusively in late spring has become ingrained in the cultural practices of grassland management in the Flint Hills, and local ranchers often burn in unison when weather conditions are favorable.

Despite long-standing recommendations that tallgrass prairie be burned only in late spring, the data supporting this policy is equivocal. Total biomass production was lower in plots burned in early spring than plots burned in late spring [19]–[21], but the weights included grasses, forbs, and shrubs. It was not known if grass biomass was reduced by early-spring burning or if the differences were a site effect rather than a treatment effect. Burning in early spring also shifted community composition in a perceived negative pattern because it favored cool-season graminoids and forbs [19], [22], [23]. This shift in community composition, however, may actually be desirable because many cool-season grasses have higher production and nutritional quality than warm-season grasses at certain times of the year [24]–[26], and many forb species are beneficial to the diet of grazers [27]–[29]. Burning in late spring has been considered the most effective time to control invasive shrubs [22], [23], but *Symphoricarpos orbiculatus* was the only woody species that declined with repeated late spring burning [19]. Finally, average weight gain of steers was lower in an unburned pasture than in burned pastures [23], but there was no significant difference in monthly weight gain among cattle grazing in early-, mid-, or late-spring burned pastures.

The historical studies that formed the foundation for time of burning recommendations in tallgrass prairie are inconclusive because none had experimental replications and most were spatially limited to small plots [19]–[23]. All of these studies were interpreted as suggesting that shifting the time of burning by only a few weeks would negatively influence the plant community. A more recent large-scale replicated study that compared the effects of annual burning in autumn, winter, and late spring found that the timing of burning had no significant effect on grass production and no reductions in the composition of desirable warm-season grasses [30]. Despite evidence that burning can occur earlier in the year without the previously predicted adverse repercussions, burning pastures only in late spring has become a firmly ensconced tradition. Arguably, the 8-year seasonal burn study could be considered an insufficient length of time to adequately represent long-term effects of burning on biomass production and plant community composition. A longer time series would span wider variations in weather and determine if response patterns from burning at different times remained consistent long-term. The study also had not examined if there are differences in the sensitivity of production to precipitation variation at different times of the year. Changes in the timing of burning could make production more susceptible to droughts or heat waves [31], increasing the risks of climate variability to ranchers. Additionally, the seasonal burn study [30] did not measure grass culm production of warm-season grasses, which can be stimulated by late-spring burning [32], and thus potentially contribute to higher grass productivity without benefitting grazers.

Our objectives in this study were to expand upon the previous 8-year data set and to more extensively test the long-term effects of burning in different seasons. Herein, we analyze 20 years of data from replicated ungrazed watersheds to test whether the timing of burning affects 1) total grass and forb biomass, 2) relationships between grass biomass production and precipitation at different times of year, 3) flowering culm production of the dominant grasses, and 4) changes in plant community composition.

## Methods

This research was conducted on and approved by Konza Prairie Biological Station, a 3,487-ha native tallgrass prairie located in northeastern Kansas (39° 05′ N, 96° 35′ W). To study how fire affects the structure and function of grassland vegetation, Konza Prairie is parceled into numerous watersheds that provide large replicated experimental units subjected to different long-term fire regimes. Topographically, the watersheds consists of shallow xeric upland soils (cherty, silty clay loams overlying limestone and shale layers; Udic Argiustolls, Florence series), and mesic lowland soils (deeper colluvial and alluvial deposits; Pachic Argiustolls, Tully series). Vegetation on both topographic positions are dominated by perennial warm-season grasses, primarily *Andropogon gerardii*, *Sorghastrum nutans*, and *Schizachyrium scoparium*, but interstitial forbs comprise more than 75% of the species richness [33]. Mean annual temperature for the area is 13°C, with mean monthly temperatures ranging from −3°C in January to 27°C in July. From 1994 to 2013, annual precipitation for Konza Prairie averaged 845 mm, with ∼73% falling in the April through September growing season. Annual precipitation ranged during this period from 581 mm in 2012 to 1,153 mm in 2008. All climate data used in this study were collected from a weather station located at Konza Prairie headquarters, ∼5 km away from the watersheds.

Six watersheds (average size = 15 ha) that had not been grazed for more than 30 years were selected for a long-term seasonal burn study. Seasonal burning began in November 1993, when two separate watersheds were burned for the autumn treatment. Subsequent fire treatments included two watersheds that were first burned in February 1994 and two more in April 1994 for the winter and spring treatments, respectively. The same two watersheds were burned in the same season throughout the study. Median burn dates for the 20-year period were November 23 for the autumn burns, February 18 for the winter burns, and April 21 for the spring burns. All burns were conducted under conditions of moderate wind speed (<6.7 m s^−1^) and humidity (40–80%), producing relatively intense head fires.

### Data Collection

Plant species composition sampling began in 1994 after four, 50-m long transects, each with five permanent plots, were established on both upland and lowland topographic positions in all watersheds (n = 20 plots for each topographic position). Every year, the canopy cover of all vascular plant species in a 10 m^2^ circular area within each plot was estimated and assigned to a percentage category [34]. Cover of individual species was determined by averaging the midpoint of the seven cover categories (i.e., 0.5, 3, 15, 37.5, 62.5, 85, and 97.5%) across the 20 plots for each topographic position. All plots were surveyed each year in early June and again in late August. For each species, the maximum cover value from the June and August surveys was used for composition analyses. No endangered or protected species were encountered in this study.

Aboveground net primary productivity (ANPP) was measured at the end of each growing season by clipping five randomly selected quadrats (0.1 m^2^) adjacent to each plant composition transect (n = 20 plots per topographic position). Vegetation in the plots was clipped at ground level, separated into graminoid, forb, and woody components, and oven dried at 60°C before weighing.

The flowering responses of the three dominant grass species were measured annually at the end of the growing season in transects adjacent to the four plant composition transects on both the upland and lowland topographic positions. Along each transect, all flowering culms that occurred in six randomly spaced 0.25-m^2^ quadrats were counted (n = 24 plots per topographic position). The flowering culms of each grass species in the plot were then harvested at ground level, dried at 60°C for 2–3 days, and weighed. In addition to annual grass production, an index of grass leaf production was determined as the difference between total grass production and culm production from the three dominant grass species for each topographic location of the six watersheds.

### Statistical analyses

To determine the effects of burning in different seasons on primary production, flowering, and species composition, data were first averaged for all plots and transects in a landscape position each year for each watershed. The response variable was then predicted with a regression model that included timing of burning, watershed nested within timing of burning as a random effect, year as a continuous variable, and all pairwise interactions. This is equivalent of a split-plot, repeated-measures, mixed-model analysis of variance [30]. Separate models were run for each landscape position. Degrees of freedom of the model were 1,111 for time, 2,3 for treatment, and 2,111 for the interaction between treatment and time. Comparisons among treatments were conducted with Tukey’s HSD.

Critical climate period analysis was used to determine the role of precipitation at different times of year on aboveground net primary productivity of grasses (ANPP_G_). For that analyses, precipitation was summed for 861 periods between day 60 (March 1) and day 274 (October 1), with a minimum length of 15 d [31], [36]. A forward stepwise regression was initiated using precipitation data from all 861 periods to explain variation in ANPP_G_ for each combination of landscape position and burn treatment. The critical precipitation period that explained the highest amount of variation in productivity was then selected as a predictor variable and the process repeated for the next most significant precipitation period. Critical climate periods of the same climate variable that overlapped in time were not allowed in the final model. For all six models, only one precipitation period was significant. Confidence intervals for the start and end dates of the critical precipitation periods were determined by calculating the mean start and end dates of the 20 date ranges that explained the most variation in grass production. To account for differences in explanatory power among date ranges when calculating the means and standard errors of critical climate period parameters in the univariate analyses, individual dates were weighted by the sum of squares explained by the date range.

Differences among burn treatments in plant community composition were assessed using non-metric multidimensional scaling (NMDS). NMDS was performed with Bray-Curtis distances of the 31 plant species that had >2% mean cover in any year or treatment. Changes in species composition over time were assessed with linear regression of cover data averaged by year for a given treatment and landscape position. NMDS analyses were carried out in R v3.0.2 using the metaMDS function of the vegan package with k = 3 and 20 random starts [35]. Results for patterns of species composition were found to be qualitative similar to those results derived from principal components analysis with Euclidean distances. All other analyses were performed in JMP 9.0.3 (SAS Institute, Cary, NC). Differences in plot scores among contrasts for each axis were assessed with least squares regression. The model included burn treatment, landscape position, year, and all pairwise interactions.

## Results

### Biomass production

**Figure 1:**
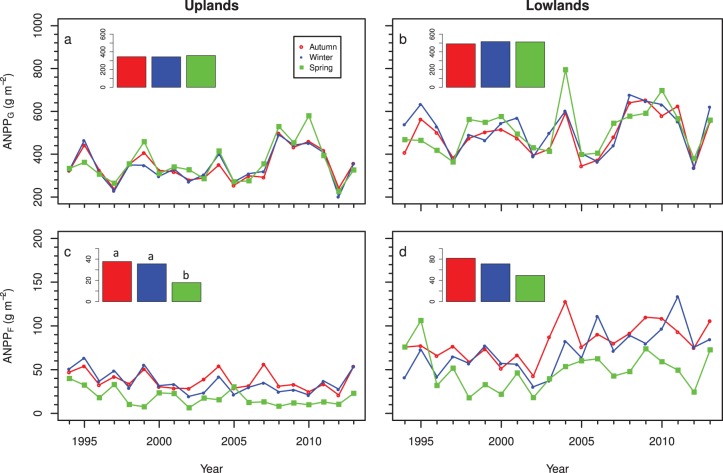
Changes in upland and lowland grass (a,b) and forb (c,d) productivity over time for autumn-, winter-, and spring-burned watersheds on upland (a,c) and lowland (b,d) positions. Inset in each graph represents the 20 year mean with means that were significantly different (*P*<0.05) denoted with different letters.

Over the 20-year study, grass production in both uplands and lowlands was correlated among all three burn times (*r>*0.67 for all paired comparisons). Across years, there was no difference in average grass production for watersheds burned in autumn, winter, or spring in either uplands (345.0, 342.9, and 360.0 g m^−2^, respectively; pooled SE = 21.0 g m^−2^; *P* = 0.83) or lowlands (491.0, 512.2, and 515.6 g m^−2^, respectively; pooled SE = 10.8 g m^−2^; *P* = 0.35; Fig. 1a, b). There also was no change in the difference in grass biomass over time between autumn- or winter-burned and spring-burned watersheds (*P>*0.44 for both landscape positions).

For uplands, forb biomass was significantly greater in autumn and winter burns than in spring burns (37.7 and 35.6 vs. 17.9 g m^−2^, respectively; pooled SE = 1.8 g m^−2^, P = 0.008), but there was no significant difference in lowlands (*P* = 0.51; Fig. 1c, d). Forb biomass decreased over time in uplands in spring- and winter-burned watersheds (0.8 and 0.9 g m^−2^ y^−1^ P = 0.02 and P = 0.009, respectively), but not fall-burned watersheds (P = 0.19). Forb biomass increased over time in lowlands for both autumn- and winter-burned watersheds (1.9±0.8 g m^−2^ y^−1^ and 2.6±0.7 g m^−2^ y^−1^, respectively; *P*<0.01 for both), but not spring-burned watersheds (*P* = 0.98). Biomass of woody species averaged 4.3 g m^−2^ across all treatments, landscape positions, and years. Woody species biomass increased 0.19±0.01 g m^−2^ y^−1^ across all treatments in uplands (*P* = 0.02), but there were no other significant differences among other contrasts.

**Figure 2:**
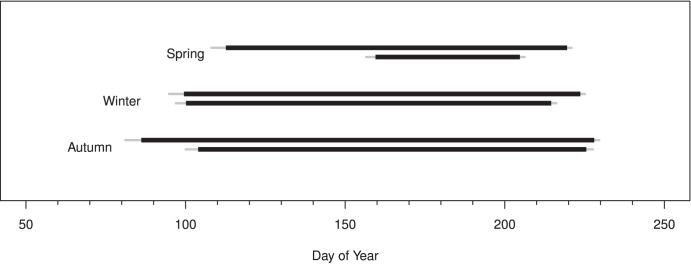
Critical climate periods for uplands (top black bar in pair) and lowlands (lower black bar in pair) for autumn-, winter-, and spring-burned plots. Gray bars represent standard errors on start and end dates for the 20 critical climate periods that explain the most variation in grass productivity.

**Figure 3:**
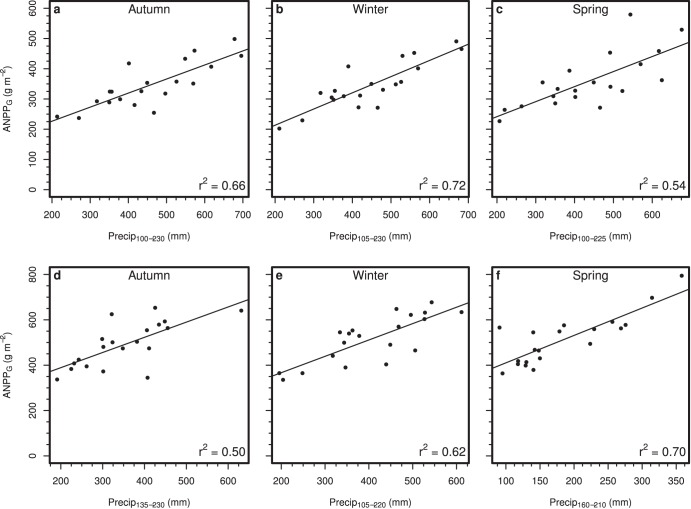
Relationships between precipitation during critical precipitation period and grass productivity (ANPP_G_) for uplands (a–c) and lowlands (d–f) for autumn (a,d), winter (b,e), and spring-burned (c,f) watersheds.

Grass production in all three burn treatments was higher in years with greater mid-season precipitation. Yet, critical precipitation periods were longer for grasslands burned in autumn or winter than spring (142±6, 124±5, and 107±5 d respectively, P<0.001 for uplands; 153±6, 124±5, 107±5 respectively, P<0.001 for lowlands; Fig. 2). Typically, grass production in watersheds burned in autumn or winter responded positively to April and May precipitation in contrast to grass production in watersheds burned in the spring. In uplands, precipitation affected grass productivity earlier in the year for autumn- and winter-burned than spring-burned watersheds (start dates DOY 86±5 and 99±5 vs. 113±5; *P* = 0.02) with precipitation affecting productivity later in the year for autumn-burned watersheds than spring-burned watersheds (end dates DOY 228±2, 224±2, and 220±2, for autumn-, winter-, and spring-burned watersheds respectively; *P* = 0.006. A similar pattern was observed for lowlands where precipitation affected grass productivity earlier in the year for autumn and winter burns than spring burns (start dates DOY 86±5 and 99±5 vs. 113±5, respectively; *P*<0.02) and later in the year for autumn burns compared to winter and spring burns (end dates 228±2 vs. 224±2 and 220±2, respectively; *P* = 0.006). On average, 63% of variation in grass productivity among years for the different combinations of landscape positions and burn treatments were explained by precipitation during the weighted average critical climate periods (Fig. 3).

**Figure 4:**
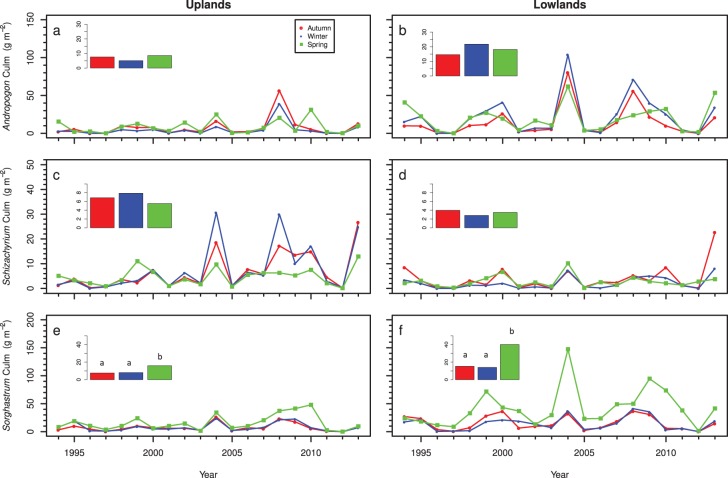
Changes in flowering culm production for (a,b) *Andropogon gerardii*, (c,d) *Schizachyrium scoparium*, and (e,f) *Sorghastrum nutans* from 1994–2013 for uplands (a,c,e) and lowlands (b,d,f) in autumn-, winter-, and spring-burned watersheds.

Although there were no significant differences in total grass biomass among treatments, culm production was higher in spring-burned watersheds than autumn- or winter-burned watersheds (*P*<0.05; Fig. 4). Culm production for autumn- and winter-burn treatments averaged 22.1±5.3 g m^−2^ and 20.4±5.1 g m^−2^ in the uplands, and 34.0±7.3 g m^−2^ and 39.2±8.8 g m^−2^ in the lowlands, respectively. In contrast, culm production in the spring-burned watersheds averaged 29.1±5.3 g m^−2^ and 64.1±11.1 g m^−2^ for uplands and lowlands respectively. The higher culm production in spring-burned watersheds was primarily due to increased *Sorghastrum nutans* flowering, which was 8.2 g m^−2^ greater in uplands and more than 26.7 g m^−2^ greater in lowlands in spring burns than the average of autumn and winter burns (P<0.05 for both comparisons). There was no significant linear increase or decrease in the difference in culm biomass among different treatments over time for either landscape position (*P*>0.9). After accounting for differences in culm production, there were no differences in grass leaf production among burn treatments for uplands (*P>*0.55 for all comparisons) or lowlands (*P>*0.11 for all comparisons).

### Species composition

**Figure 5:**
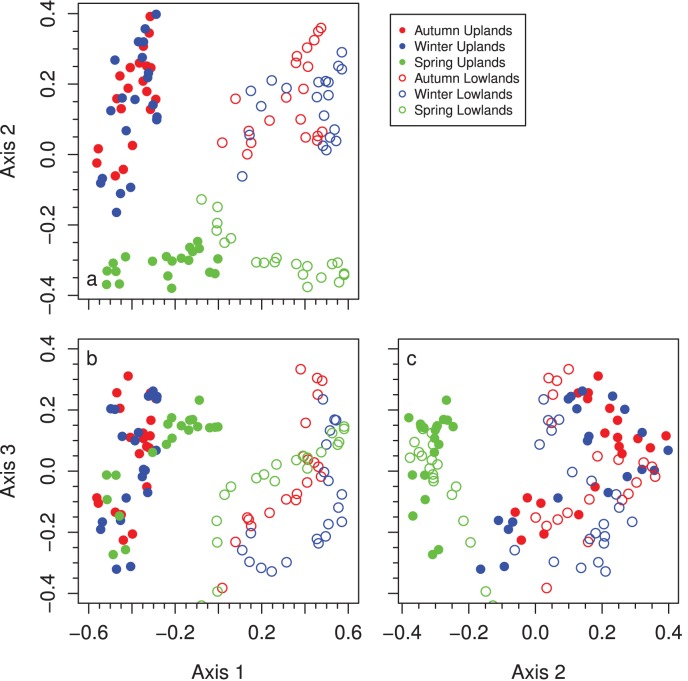
Relationships among NMDS axes of upland (closed circles) and lowland (open circles) grasslands burned in autumn (red), winter (blue), or spring (green). Stress value = 0.07 for k = 3.

**Figure 6:**
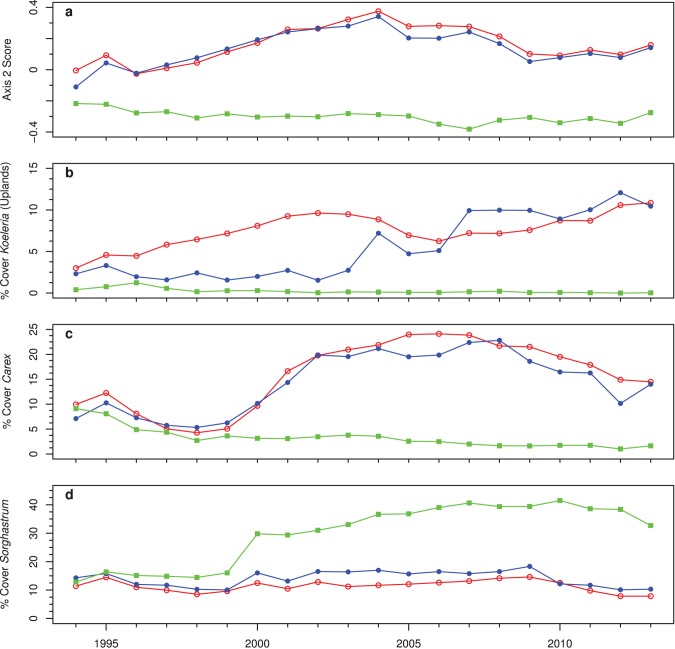
Changes from 1994–2013 of (a) NMDS axis 2 scores for all upland and lowland plots, and cover values of (b) *Koeleria macrantha*, (c) *Carex* spp., and (d) *Sorghastrum nutans*. Cover values were averaged for uplands and lowlands for a given burn treatment for all examples, except for *Koeleria*, which is shown for just uplands, since it was rarely found in lowlands.

Changes in species composition in response to the timing of burning reveal that autumn and winter burns promote a broader phenological diversity of species than annual spring burns (Table 1). The second NMDS axis primarily separated spring-burned watersheds from those burned in autumn or winter (r^2^ = 0.79, P<0.001; Fig. 5) and the differences between these treatments increased over time for both landscape positions (*P*<0.01; Fig. 6a). Spring-burned watersheds had higher cover of *Sorghastrum nutans*, *Ruellia humilis*, *Asclepias viridis*, and *Bouteloua curtipendula,* while autumn- and winter-burned watersheds had higher cover of *Symphyotrichum ericoides*, *Symphyotrichum oblongifolium*, *Koeleria macrantha*, *Dalea candida*, and *Carex* spp. (predominantly *C. inops* and *C. meadii*). Among the graminoids, *Koeleria macrantha* and *Carex* increased the most in abundance with autumn or winter burning (Fig. 6b, c). *Koeleria macrantha*, a species that predominantly occurs in uplands, increased from an average of 3% to 11% in autumn- and winter-burned watersheds, but was extirpated in plots burned in the spring. *Carex* cover increased from an average of 8% to 14% in the autumn- and winter-burned watersheds, but decreased from 6% to 1% in spring-burned watersheds. In contrast, *Sorghastrum nutans* cover increased greatly with spring burning compared to autumn or winter burning (Fig. 6d). With annual spring burning, *Sorghastrum nutans* cover increased from 12% to 32% in uplands and 14% to 52% in lowlands. With annual autumn or winter burning, *Sorghastrum nutans* cover essentially remained constant at 13%. Cover of woody species did not different among burn treatments (P>0.05).

**Table 1:** Scores of species for the first three axes of non-metric multidimensional scaling analysis generated from the 31 most abundant species among the six watersheds of the study.

*Ruellia humilis*	0.17	−0.44	−0.17
*Sorghastrum nutans*	0.18	−0.38	0.03
*Asclepias viridis*	0.13	−0.36	−0.16
*Bouteloua curtipendula*	−0.73	−0.31	0.09
*Physalis pumila*	−0.08	−0.22	−0.42
*Brickellia eupatorioides*	−0.59	−0.20	−0.29
*Salvia azurea*	−0.75	−0.16	0.07
*Amorpha canescens*	0.13	−0.15	−0.20
*Panicum virgatum*	0.46	−0.12	0.18
*Solidago missouriensis*	0.23	−0.07	−0.09
*Dalea purpurea*	−0.54	−0.05	0.05
*Rhus glabra*	0.42	−0.02	−0.36
*Andropogon gerardii*	0.07	−0.02	−0.02
*Sporobolus heterolepis*	−0.51	−0.01	0.11
*Lespedeza capitata*	0.49	0.00	−0.18
*Poa pratensis*	0.02	0.01	−0.67
*Solidago candensis*	0.72	0.02	−0.01
*Schizachyrium scoparium*	−0.45	0.03	0.22
*Lespedeza violacea*	0.80	0.04	0.17
*Sporobolus compositus*	−0.03	0.07	−0.50
*Vernonia baldwinii*	0.13	0.07	−0.22
*Psoralidium tenuiflorum*	−0.11	0.15	0.04
*Ambrosia psilostachya*	0.33	0.18	−0.01
*Dichanthelium oligosanthes*	−0.52	0.21	−0.07
*Artemisia ludoviciana*	0.05	0.30	0.06
*Baptisia bracteata*	−0.31	0.35	0.07
*Carex spp*	−0.11	0.37	0.10
*Koeleria macrantha*	−0.83	0.45	0.40
*Dalea candida*	0.59	0.45	−0.14
*Symphyotrichum oblongifolium*	−0.84	0.48	0.20
*Symphyotrichum ericoides*	0.18	0.52	−0.39

Axis 2 was most associated with timing of burning with autumn- and winter-burned treatments scoring high on the axis and spring-burned watersheds scoring low.

The other two multivariate axes were primarily associated with differences in composition between landscape positions and general trends in composition with time independent of burning. The first axis primarily separated uplands and lowlands in their composition (r^2^ = 0.81, *P*<0.001; Fig. 5). Upland plots had greater cover of *Symphyotrichum oblongifolium*, *Koeleria macrantha*, *Salvia azurea*, *Bouteloua curtipendula*, and *Brickellia eupatorioides* than lowland plots (Table 1). Lowland plots had greater cover of *Solidago canadensis, Lespedeza violacea*, *Lespedeza capitata*, *Dalea candida*, and *Panicum virgatum* relative to upland plots (Table 1). The third axis primarily represented declines in some species that occurred over time irrespective of burn treatment and topographic position (r^2^ = 0.76, *P*<0.001; Fig. 5). For example, *Sporobolus compositus,* which loaded strongly on Axis 3 (Table 1), declined from an average of 5.2% cover in 1994 to 0.1% in 2013.

## Discussion

Results from this study do not provide any compelling reason to wait until late spring to burn Flint Hills grasslands. Three lines of evidence support the premise that tallgrass prairie can be burned earlier in the year with little adverse effect. First, grass biomass was not significantly different in grasslands burned in autumn, winter, or spring, even though spring-burned grasslands did produce more low-quality grass stems. Second, grass productivity in spring-burned grasslands relies on precipitation during a narrower window of time during the growing season than autumn- or winter-burned grasslands, which would increase the likelihood of severe consequences from summer drought. Lastly, the increase in abundance of cool-season grasses and forbs without a decline in warm-season grasses would benefit grazers by providing high-quality forage both earlier in spring and later in autumn. This potentially would allow for more consistent, if not earlier, stocking of steers, and also may allow cattle in season-long grazing systems to remain on pasture for a longer time in autumn.

The general patterns of early-season burning promoting cool-season graminoids and some forb species without reducing overall productivity were also observed previously after 8 years of differences in seasonal timing of burns [30]. As such, while the shifts in species composition became more pronounced over time, the lack of differences in production was not a transient effect. The finding that late-spring burns promote the flowering of a dominant warm-season grass has been previously noted with *Andropogon gerardii.* Burning after foliage production began was associated with up to three times greater flowering density than burning before initiation of foliage production [32]. Although we did not detect any difference in *Andropogon gerardii* flowering among the burn treatments, we did find that *Sorghastrum nutans* flowering is strongly stimulated by late-spring burning but not autumn or winter burning. Greater phenological diversity in autumn and winter burned areas compared to spring burned areas was either due to winter and autumn burns directly promoting forbs and cool-season graminoids or injuring them less than spring burns. Early growing species are particularly susceptible to late-spring burns. For example, *Carex* and *Koeleria macrantha* begin flowering in late-April or early-May [26] and their canopy covers were severely reduced with late-spring burning, but gradually increased from burning at other times.

One mechanistic reason that burning in early spring has been discouraged is because the removal of protective litter would supposedly increase evaporation and reduce water infiltration [37], [38]. The reduced soil moisture would subsequently lower grass production compared to unburned or late-spring burned plots [23], [39]. However, data from those studies actually indicate that soil moisture levels in early-spring and late-spring burns declined at similar rates over time, suggesting that time of burning was not differentially affecting soil moisture losses. We did not measure soil moisture, but since total grass production did not differ among burn treatments in either topographic position, it is doubtful that soil moisture levels were substantially different [36], [40]. Nevertheless, future research should address seasonal patterns of evapotranspiration and soil moisture associated with differences in the timing of burning to definitively resolve the issue.

Currently, the emphasis on burning only in late spring is based on its potential effects on cattle production and purported enhanced control of woody species. However, since the only study that measured cattle performance lacked spatial replication and did not show any significant differences in monthly weight gain among early-, mid-, and late-spring burns when years were used as replicates [23], the contention that early season burning negatively affects animal performance needs to be objectively reevaluated. Additionally, we found no differences among burn treatments in woody species canopy cover, suggesting that late spring burning was not inherently superior in controlling woody species. Burning earlier in the season allows more flexibility in the date that cattle are stocked and may be beneficial by allowing earlier stocking when nutritional quality of cool-season grasses is high. In addition, there has never been a plausible explanation for why tallgrass prairie should be burned once new growth of the dominant grasses are 1.25–5 cm tall [10], [41]. This early-season growth is highly nutritious for cattle [42] and removing it by burning in late spring represents lost productivity and nutritional quality. Burning pastures without being restricted to a narrow window in late spring also offers ranchers greater flexibility in ensuring that the area gets burned. For example in years with above average April temperatures or precipitation, the vegetation may quickly progress to where it becomes unfeasible to burn. Lastly, it typically requires 10–14 days after a late-spring burn before there is sufficient grass growth to support grazing, thereby delaying when animals can be released on pasture compared to vegetation in pastures burned earlier in the spring.

Shifting burning from late spring to earlier times in the year could mitigate concentrated smoke pollution and reduce the likelihood of exceeding ozone levels downwind. The presence of high-moisture, nitrogen-rich grass during late-spring burns reduces the completion of combustion and increases NO_x_ formation [18]. Tropospheric ozone is not monitored in Kansas cities from November through March because the lower temperatures and insolation during the autumn and winter reduces the probability of high ozone concentrations being formed [43]. Thus, pasture burning at those times likely would help alleviate air quality issues. Historically, fires in North American prairies also occurred in summer [44], but prescribed fires during these times, even if they were to be considered by managers, would only exacerbate smoke management issues.

An additional concern about grassland burning for many landowners is the impact that it may have on indigenous wildlife, Ground-nesting birds have often laid eggs by late-April, and clutches of early-nesting birds, such as Greater Prairie-Chickens (*Tympanuchus cupido),* are particularly susceptible to late-spring burning [45]–[47]. Snakes, tortoises, and other vertebrates also are more active in late spring than in early spring and thus are at greater risk from fires occurring in late-spring [48], [49]. Although we did not measure wildlife casualties, burning earlier in the spring would be less likely to negatively impact ground dwelling species.

## Conclusion

The current balance of scientific research provides little support for the recommendation that ranchers should wait until late spring to burn grasslands in the Flint Hills. Although this research was conducted on ungrazed watersheds where fire intensity is greater than in grazed pastures, grazing can interact with burning and possibly alter some of the observed response patterns. Consequently, there is a need for research that specifically examines the response of plant production in grazed watersheds to burning at different times of the year. Current evidence indicates, however, that grass productivity from burning early in the year will be just as high as burning in late spring with no negative impacts on desirable grass species. Grasslands burned early also would be less impacted by mid-summer droughts, the greater plant phenological complementarity would allow for more flexible stocking dates, wildlife such as reptiles and ground-nesting birds would be less likely to be negatively impacted, and ranchers would gain flexibility in scheduling burns. In addition, burning before April should reduce the risk of exceeding air quality standards from smoke production.

This 20-year study indicates that burning before late spring is a sustainable management practice with little apparent negative repercussions relative to burning in the late spring. There is, however, a need for a broader distribution of measurements that include cattle weight gain to test for patterns across the entire Flint Hills region. This likely would not be difficult to implement since most ranchers already weigh cattle at the beginning and end of the grazing season. As such, altering the timing of burning and measuring weight gain for extant operations would provide a broad dataset on the timing of burning and cattle weight gain. In other grasslands, the timing of burning is just as relevant as in the Flint Hills because the need to manage smoke production is an issue wherever prescribed burning occurs. But any policy recommendations on timing of burning should ultimately be based on long-term replicated studies to fully understand the consequences of burning at different times.
